# Low-Fidelity, In Situ, Accessible Pediatric Mass Casualty Incident Simulation to Evaluate and Improve Pediatric Readiness

**DOI:** 10.15766/mep_2374-8265.11538

**Published:** 2025-06-27

**Authors:** Sydney E. Jeffs, Cathlyn K. Medina, Parker Frankiewicz, Steven W. Thornton, Elizabeth Horne, Smith Ngeve, Tara Thomason, Delaney Anani-Wolf, Catherine B. Beckhorn, Delaney James, Rachel Hobbs, Remi Hueckel, Corrie E. Chumpitazi, Erin R. Hanlin, Rachel O'Brian, Elisabeth T. Tracy, Emily Greenwald

**Affiliations:** 1 Medical Student, Duke University School of Medicine; 2 Surgery Resident, Department of Surgery, Duke University School of Medicine; 3 Surgery Resident, Johns Hopkins Hospital; 4 Medical Student, University of North Carolina at Chapel Hill School of Medicine; 5 Nurse Manager, Department of Emergency Medicine, Duke University Hospital; 6 Nurse Practitioner, Department of Emergency Medicine, Duke University Hospital; 7 Associate Professor of Nursing, Division of Pediatric Critical Care, Duke University School of Nursing; 8 Professor of Pediatrics, Division of Pediatric Emergency Medicine, Department of Pediatrics, Duke University School of Medicine; 9 Assistant Professor of Emergency Medicine, Department of Emergency Medicine, Duke University School of Medicine; 10 Assistant Professor of Pediatrics, Division of Pediatric Emergency Medicine, Department of Pediatrics, Duke University School of Medicine; 11 Pediatric Surgeon, Division of Pediatric Surgery, Department of Surgery, Duke University School of Medicine; † Co-primary author

**Keywords:** Simulation, Pediatrics, Mass Casualty Incident, Triage, Communication Skills, Interdisciplinary Medicine, Interprofessional Education, Case-Based Learning

## Abstract

**Introduction:**

Existing mass casualty incident (MCI) simulations rely on high-fidelity patient simulators, which are cost-prohibitive and often exclude pediatric patients. To address the need for deployable, low-fidelity pediatric MCI simulations, we developed and evaluated a cost-conscious model to teach the principles of JumpSTART, the pediatric variation of the Simple Triage and Rapid Treatment (START) algorithm.

**Methods:**

In this low-fidelity pediatric MCI simulation, pediatric trauma patients were represented by 2D, life-sized drawings including all pertinent information for triage using JumpSTART. Learners were prehospital and hospital staff with multidisciplinary backgrounds. Learners were divided into two groups and assigned five unique patients across triage and acuity levels. Primary outcomes were the accuracy of assigned triage categories and Broselow lengths, and time to triage completion. Postsimulation surveys were designed to assess learner attitudes about the exercise.

**Results:**

Two sessions of the pediatric MCI simulation were conducted (18 and 16 participants, respectively). Triage categories were correctly assigned using JumpSTART for 9 of 10 patients in cohort 1. One patient was over-triaged. All patients in cohort 2 were correctly assigned triage categories. Broselow lengths were correctly assigned to all patients. Median time to assign a triage category per patient was 67 seconds (range 30–135) for the first cohort and 64 seconds (range 30–116) for the second. Participant feedback was universally positive.

**Discussion:**

We present an accessible, low-fidelity training model for pediatric MCI, which creates a simple but dynamic hands-on experience for participants around the JumpSTART pediatric triage algorithm and is replicable across environments.

## Educational Objectives

By the end of this activity, learners will be able to:

1. Integrate knowledge of pediatric physiology and triage tools, including the Broselow tape and the JumpSTART triage method, to accurately assign triage categories and direct management in a pediatric mass casualty incident.
2. Communicate effectively between prehospital and hospital staff on interdisciplinary care teams to ensure consistency in care transitions and maintain ongoing evaluation and management.
3. Apply dynamic triage in a mass casualty event to assign initial triage categories, reevaluate patients, prioritize interventions, and allocate resources efficiently across multiple patients with varying acuity levels.
4. Develop skills to recognize and manage emotional responses when treating patients in pediatric mass casualty incidents, understanding their impact on decision-making.

## Introduction

A mass casualty incident (MCI) is defined as “an event that overwhelms the local healthcare system, where the number of casualties vastly exceeds the local resources and capabilities in a short period of time.”^1^ Children are a particularly vulnerable population in disasters given their anatomic, physiologic, immunologic, and developmental vulnerabilities.^2,3^ There is often a knowledge gap among emergency responders in the prehospital and hospital environments regarding injury patterns unique to pediatric patients and the physiologic response to injury/trauma, which supports a need for pediatric-focused teaching and preparation.^4,5^ While pediatric MCI events may be rare, when they do occur, it is vital that the multidisciplinary teams responding to them are prepared to appropriately account for pediatric physiology to accurately triage and prioritize resources during these high-stakes events.^6^

Simulations are an established educational paradigm that are ideally suited to prepare learners for high-acuity, low-frequency events and have been demonstrated to result in a stronger, longer-lasting learning experience for participants in comparison to strictly didactic methods.^7,8^ Current published work discussing MCI simulation models primarily focuses on the triage and care of adult patients.^9–11^ These models often use manikin-based simulators or standardized patient models with moulage. These high-fidelity MCI simulations are time-intensive and involve planning and utilizing resources that are not broadly accessible across academic and nonacademic centers.^12–15^ While some groups have demonstrated successful pediatric MCI simulations with high-fidelity resources,^16,17^ there remains a dearth of literature describing inexpensive, accessible alternatives that allow for deliberate practice of specialized skills and integration of knowledge essential for the care of pediatric patients.

To address the limitations of high-cost, resource-intensive simulation modalities and improve accessibility, we designed a low-fidelity, in situ pediatric MCI simulation model using 2D patient representations.^16^ We developed a curriculum to determine the feasibility of implementing this simulation within our institution, measured by successful completion of the simulation by participant cohorts within a reasonable timeframe. We also aimed to assess the efficacy of the model, as indicated by participant performance in applying a pediatric-specific adaptation of the Simple Triage and Rapid Treatment system (JumpSTART) and Broselow tape, and by participant perceptions of the simulation's utility.

## Methods

### Development

Previous pediatric simulations at our institution primarily involved single-patient trauma scenarios, emphasizing iterative improvement and rapid-cycle accuracy.^18^ These efforts led to the development of the Trauma Cognitive Aid structured observational tool for trauma resuscitation, now standard in all pediatric trauma bays, demonstrating the impact of simulation training on institution-wide intervention.^19^ Our multidisciplinary team with expertise in pediatric trauma and simulation then designed an innovative curriculum using low-fidelity, 2D patient models to improve learner confidence in rapid, accurate triage of multiple pediatric MCI patients. This project was deemed exempt by the Duke University Health System Institutional Review Board (Pro00109569).

We designed the simulation environment for the ambulance bay to emphasize elements of the transition of care from the prehospital to hospital environment, such as interdisciplinary coordination and dynamic triage. Unlike our prior single-patient simulations, here we chose a multiple-patient model incorporating mass casualty complexity and resource allocation decisions. Using an adaptation of Tan et al.'s simulation and incorporating previously published triage assignments,^16^ we included as cases patients ranging in age from 6 months to 17 years who varied in size and type of traumatic injury, to reflect real-world unpredictability. Our use of adult-sized teenage patients highlighted distinctions between adult and pediatric triage.

We emphasized the importance of pediatric-specific decision-making by utilizing the JumpSTART pediatric triage algorithm and the Broselow tape for determining size-based interventions.^16^ By implementing the Broselow tape, we provided learners a method to make quick and accurate estimations of a child's weight based on their height, which is crucial in emergency situations for quickly determining appropriate medication dosages, equipment sizes, and fluid volumes. Learners in Team A assessed patients, determined Broselow lengths, assigned triage categories, and handed off to learners in Team B with a summary of findings and intervention instructions. Team B then discussed prioritization for emergent interventions across patients in a resource-strained setting.

Facilitators, including a pediatric emergency medicine attending physician and a pediatric surgeon, were directly involved in the preparation of patient scenarios and materials, and therefore had a thorough understanding of the JumpSTART algorithm. To ensure consistency and preparedness, all facilitators were provided with the materials to conduct the simulation exercise, including the patient narratives and JumpSTART, 1 week prior to the first simulation. Participants included surgery and emergency medicine residents, pediatric emergency medicine nurses, transport paramedics, emergency department technicians, respiratory therapists, and students. Postsimulation debriefs followed the gather, analyze, and summarize (GAS) framework, focusing on correct use of the JumpSTART algorithm, Broselow tape, and continuous re-triaging.^20,21^ An anonymous survey was used to evaluate team dynamics and the perceived utility of JumpSTART, and results are reported as median Likert-scale ratings. No prerequisite knowledge was required to participate.

### Equipment/Environment

Our multidisciplinary pediatric MCI simulation was conducted in the ambulance bay of the Duke University emergency department but can easily be adapted to other environments, including a classroom or simulation lab (Appendix A). Participants were divided into two groups, evenly balancing training level and professional background. Each group was split into Team A (relaying) and Team B (receiving). A scenario introduction (Appendix B)^16^ was read to orient participants to the setting and available triage tools. Posters of both the JumpSTART algorithm (Appendix C) and the trauma cognitive aid (Appendix D) were displayed in the triage area. Two tables served as triage stations, and 10 low-fidelity, 2D paper patient models, including all necessary triage information, were used for in situ implementation (Appendix E). Digital templates of the 2D patients were created for easy distribution and case modularity (Appendix F). Each team used Broselow tapes to assign Broselow lengths, and used sticky notes to record triage categories, lengths, and next steps for interventions. Items and costs are detailed in Appendix G. Facilitators had a master list of patient presentations (Appendix H) to address any participant questions. The simulation workflow is represented in Appendix I. Coordinators used a data sheet (Appendix J), pen, and stopwatch or cell phone to record results.

### Personnel

A minimum of four participants are needed to form a two-person relaying team (Team A) and a two-person receiving team (Team B) to triage the simulation patients. Multiple groups can run through the simulation in parallel. Participants can include any team member regardless of prior knowledge or skills. We utilized two faculty members, one pediatric emergency medicine attending physician and one pediatric surgeon, to facilitate orientation and postsimulation debriefing. During the simulation, only one facilitator is needed per group. We ran two groups during each of the two MCI sessions. Facilitators should be familiar with the JumpSTART pediatric triage algorithm; experience with previous MCI or simulation curricula is beneficial but not required. A trained simulation educator can enhance the team by ensuring adherence to best practices. Additionally, two logistics coordinators are needed to record data for each team. Training fidelity is enhanced by including clinicians and learners with diverse training backgrounds, including prehospital experience. Other institutions can adapt the team composition to reflect local resources and expertise.

### Implementation

Learners were recruited via emergency department and general surgery departmental listservs. Simulations occurred during protected resident education time in the ambulance bay. The entire process, including the introduction, learner organization, debrief, and survey administration, was designed to fit within 1 hour. Setup was completed 15 minutes prior to the simulation. The first 10 minutes were for gathering participants, followed by 10 minutes for orientation, prebriefing, and team organization, 20 minutes for the simulation, 15 minutes for debriefing, and 5 minutes for postsimulation surveys. Debriefing was conducted at the simulation site.

Participants were split into groups of 8–9 learners, which were further subdivided into Team A (relaying) and Team B (receiving), comprising 4–5 participants each. Two triage stations were created with five 2D patients stacked on each table stretcher and assigned to the receiving teams of each group. Patient stacks represented various triage categories (Appendix H), including at least one patient with no significant chance of a successful outcome who would be categorized as expectant under conventional JumpSTART triage categories (Appendix C).^22,23^ Facilitators oriented both teams. Team A leaders, identified by learner consensus, led the triage, starting with Broselow tape measurements. Findings on mental status, respiration, pulse, and injuries were communicated within the team. Following deliberation and consensus, a JumpSTART triage category was recorded on sticky notes. The leader of Team A communicated their findings to Team B, which reviewed the trauma assessment and prioritized emergent interventions. Once the patient was handed off to the receiving team, Team A reinitiated the process with the subsequent patient in the stack. This process continued until all five patients had been assessed and triaged. Logistics coordinators collected data on triage category, Broselow length, decision time, discrepancies, resource allocation, and intervention priorities.

Following the conclusion of the simulation exercise, facilitators conducted a debrief focused on appropriate use of the JumpSTART pediatric triage algorithm and Broselow tape. Facilitators, who were experienced clinicians and simulation educators, were prepared to support learners in processing these emotions, though no formal psychological support was provided. A brief, internally developed survey regarding team dynamics and simulation utility was administered at the end of each simulation (Appendix K). This survey was developed and revised by the project authors, who prioritized assessment of feasibility and efficacy.

### Debriefing

The postsimulation debriefing exercise was designed for learners to participate in self-reflection and assess strengths and areas of weaknesses identified amongst themselves during the scenario. Facilitators guided this process by posing targeted reflective questions such as: “What went well?”; “How did you feel during this scenario?”; “What could have gone better?”; and “How did your team function during the scenario?”.

Facilitators encouraged open discussion of emotional responses and decision-making challenges, particularly regarding resource allocation and expectant triage. Facilitators drew on their clinical and simulation experience to support learners in processing these emotions. After a brief guided discussion, facilitators synthesized group observations with their own expert insight into strategies and pitfalls for effective communication and management during initial evaluation and triage assessment, patient hand-off, and discussions of resource allocation.

Each team was then asked to present patients they felt were challenging to triage or to identify areas of disagreement that arose within the group when assigning a triage category. Teams were prompted to outline how they might prioritize their respective patients for emergent interventions, such as which patients should go to the operating room in a resource-limited setting. Facilitators prompted learners to reflect on patient assessment and prioritization with questions such as: “How did you feel when faced with the decision to assign a pediatric patient to the ‘expectant’ category, and what factors influenced that decision?”; “How did you navigate those emotions in your decision-making?”; “How did or would you approach prioritizing your patients for the next emergent interventions?”; and “What factors would guide your decision-making process?”. Where disagreement was noted within or across teams during the triage process, these points were revisited with both reflective and focused questions such as: “I notice a few discrepancies during the triage process. I'm curious to hear how the team navigated those discrepancies” and “What rationale would inform the order in which patients should go to the operating room?”.

The guided discussion format combined self-reflection with didactic insight from content experts and encouraged participants to synthesize their experiences during the simulation to solidify learned skills and mental preparedness should they be involved in patient care following a real-life MCI. The need for continued evaluation and assessment in the triage environment was emphasized throughout the discussion. The debriefs also allowed for discussion surrounding the emotional aspects of prioritizing limited resources among pediatric patients. Debriefs were concluded by reiterating the educational objectives and any final questions were answered.

### Assessment

We assessed the feasibility and efficacy of a low-fidelity, in situ simulation designed to improve pediatric MCI preparedness. Feasibility was assessed by determining participants’ ability to successfully conduct the simulation with interdisciplinary teams within a 60-minute timeframe and by documenting any logistical challenges encountered. Efficacy was evaluated based on participant performance in applying the JumpSTART algorithm and Broselow tape, as well as participant perceptions of the simulation's utility, relevance, and impact on their MCI preparedness. Efficacy was measured by accuracy of triage category assignment, correct use of Broselow tape, and time to completion, as recorded by logistics coordinators. A QR code linked to an electronic survey (Appendix K) was distributed at the end of each simulation to anonymously gather participant feedback on team dynamics and the simulation's perceived utility.

## Results

Since developing this model, two MCI simulations have been conducted over a 3-month period. The two cohorts were kept separate to facilitate interdisciplinary team dynamics, ensure adequate hands-on experience at the triage stations, and assess intersession variability. Although the groups were run at separate times, the experiences were standardized, meaning that the results were similar between the two groups. The first simulation included 18 multidisciplinary participants (RN, 56%; resident, 22%; paramedic, 17%; respiratory therapist, 5%), and the second included 16 (RN, 44%; resident, 31%; medical student, 19%; ED tech, 6%).

Across the two cohorts of learners, only 3 participants self-identified as having familiarity with the JumpSTART triage algorithm at the start of the simulation exercise. The first cohort correctly used the JumpSTART triage algorithm to assign appropriate triage categories to nine of 10 patients, with one patient being over-triaged. The second cohort correctly assigned triage categories to all 10 patients (Figure). Both cohorts accurately measured and assigned Broselow lengths to all patients.

**Figure.**
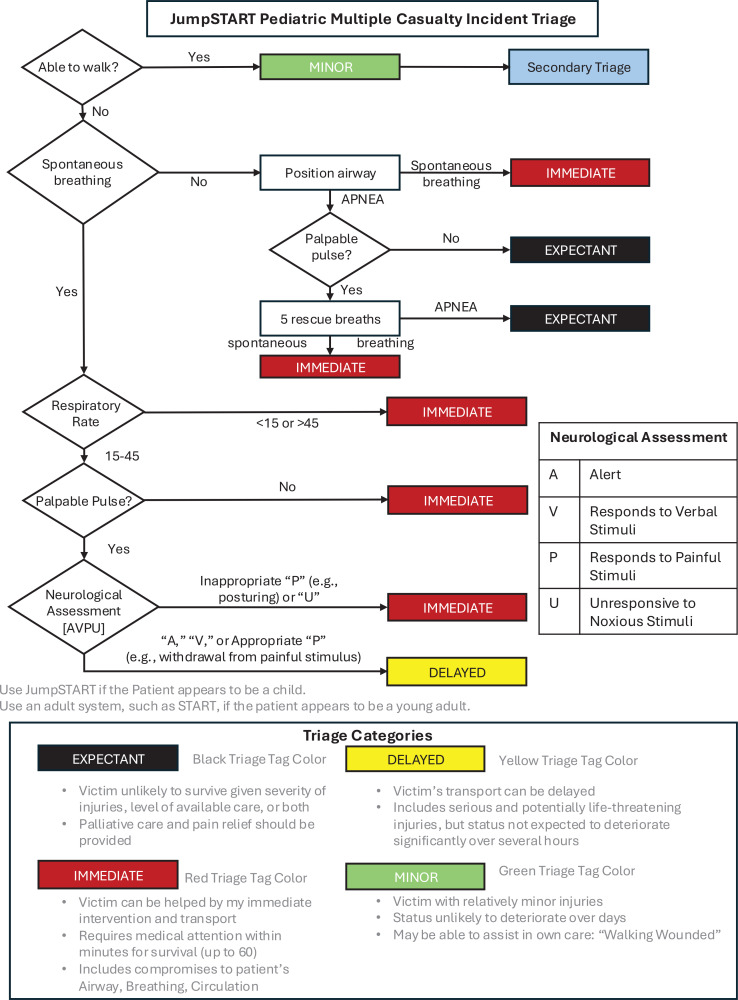
Correct triage category assignment by tag color using the JumpSTART pediatric mass casualty incident triage algorithm (see Appendix C). Abbreviation: JumpSTART, pediatric-specific adaptation of the Simple Triage and Rapid Treatment system.

The median time to assign a triage category and communicate pertinent findings to the receiving team was 67 seconds per patient (range 30–135 seconds) for the first cohort and 64 seconds per patient (range 30–116 seconds) for the second cohort (Table 1). Unfortunately, accurate times for patients 9 and 10 in the first cohort could not be obtained because of equipment malfunction, and therefore these patients were omitted from the triage time analysis. In published studies of mass casualty triage, the average reported triage times per patient ranged from 30 seconds to 120 seconds, depending on scenario complexity and learner experience.^17,24^ Our median triage times of 64–67 seconds per patient are consistent with these published standards.

**Table 1.**
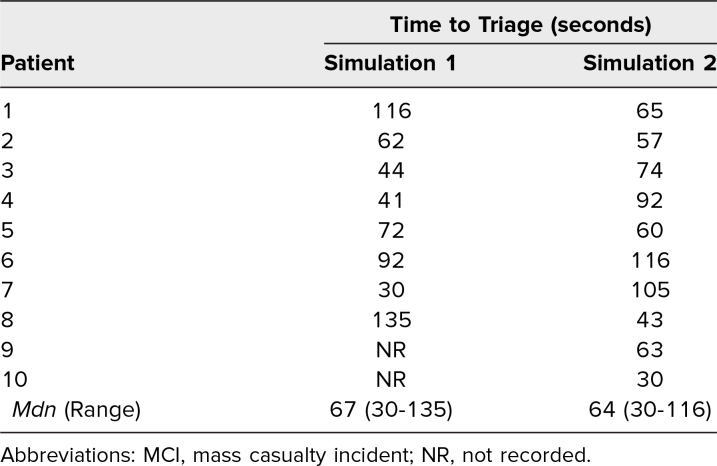
Time to Reach a Triage Decision per Patient During the MCI Simulation

Most participants who completed the survey indicated that their teams worked together effectively and performed well overall during the simulation (Table 2). The majority of learners also agreed that using the JumpSTART triage algorithm helped reduce variability and improve efficiency during triage assessments. Free responses from participants were generally positive, describing the simulation as “educational” and having a “broad application to health profession education.” Suggested areas for improvement included increasing the frequency of simulations, allocating time to review each individual patient case to reinforce the triage algorithm, and providing “a little more information” about depicted injuries. Additionally, the debriefs included discussions on the imperfect nature of triage algorithms and the necessity of using clinical judgment in tandem with triage tools. For instance, in accordance with the triage algorithm, a patient with a broken ankle would be triaged into the delayed category (yellow) due to their inability to walk, but their hemodynamic status might have them triaged more appropriately into the minor category (green).

**Table 2.**
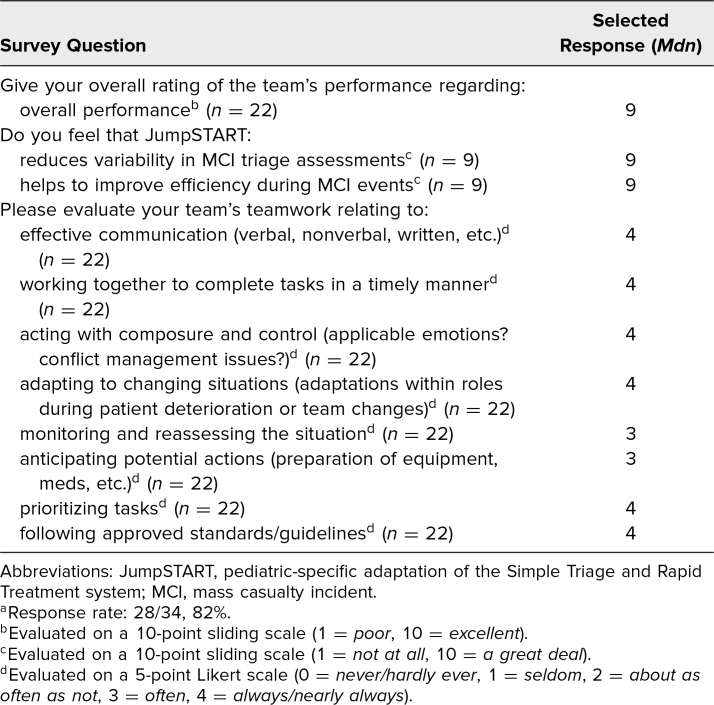
Mean Survey Scores Rating Team Dynamics and Utility of the JumpSTART Triage Algorithm (*N* = 28)^a^

## Discussion

This project contributes to the literature on pediatric MCI preparedness by demonstrating a cost-effective ($300), low-fidelity simulation model using life-sized, 2D pediatric patient replicas. This scalable approach, supplemented by customizable digital patient files, addresses resource limitations faced by many institutions. The project also underscores pediatric-specific challenges, such as the use of the JumpSTART pediatric triage algorithm and Broselow tape, which differentiates this model from existing adult-focused simulaitons.^24^

A primary education objective of this simulation was to teach and reinforce the correct application of the JumpSTART algorithm and Broselow tape for pediatric triage. Learner performance, as measured by accuracy of tirage category and Broselow length assignments, demonstrated effective acquisition of these skills. Although longitudinal data on this low-fidelity simulation model remain limited and we are unable to provide pre- and postperformance data, the positive feedback from participants regarding their learning experience demonstrates the value of this intervention. Furthermore, recorded triage decision times align with those from high-fidelity simulations,^25^ suggesting that our simulation provided a realistic and appropriately challenging environment for learners.

Discussions revealed that few learners had prior experience with the JumpSTART algorithm, underscoring the importance of these simulations for introducing standardized, evidence-based care algorithms. Our stated objective of developing learner skills in recognizing and managing emotional responses when treating patients in pediatric MCIs was not a directly measured outcome. However, structured debriefings provided a methodology for reflection and reinforced discussions on both emotional responses and the balance between clinical judgment and algorithmic decision-making. Unlike adult-focused MCI simulations that emphasize triage efficiency, this work captures the psychological complexities of pediatric triage.^26–28^

Despite the valuable insights gained, limitations exist. While participant feedback was robust, there are inherent limitations to using Likert-scale ratings, and quantitative measures of knowledge retention, skill development (e.g., interdisciplinary communication), and implementation were lacking—a common challenge in training for rare events. Future iterations could incorporate pre- and postsimulation surveys to assess educational objectives and measure the durability of learning outcomes. Lastly, learners suggested that all patients be reviewed during the debrief session to reinforce the triage algorithm, and that the frequency of simulation implementation be increased.

While high-fidelity models require substantial resources, this low-fidelity approach prioritizes accessibility and scalability, enabling institutions with limited funding to implement pediatric-specific training.^25^ Digital patient files can be printed at scale, with dry-erase lamination allowing case scenario modifications. Frequent implementation across diverse learner groups could optimize preparedness. Although more advanced tools are employed in real-life MCI situations, this simplified model facilitates widespread adoption while reinforcing core pediatric triage principles.

Future studies could compare this model to high-fidelity simulations and further explore the unique needs of pediatric patients during MCIs. Additionally, integrating first responders into these simulations could enhance realism and strengthen community-wide pediatric trauma preparedness. Participants valued the opportunity to manage multiple patients and allocate resources dynamically in a high-acuity setting. This project builds on previous work emphasizing individual resuscitation efficiency, highlighting the multidimensional nature of pediatric trauma care.

## Appendices


Implementation Guide.docxPediatric Mass Casualty Incident Simulation.docxJumpSTART.docxTrauma Cognitive Aid.docxLayout for In Situ Implementation.docxDigitized Patient Templates for Distribution.docxMaterial Costs.docxPatient Presentations.docxPediatric MCI Simulation Workflow.docxSimulation Data Collection Sheet.docxPostsimulation Survey Questions.docx
*All appendices are peer reviewed as integral parts of the Original Publication.*
