# Supplementary material for: Low-Fidelity, In Situ, Accessible Pediatric Mass Casualty Incident Simulation to Evaluate and Improve Pediatric Readiness
Source: MedEdPORTAL. 2025 Jun 27;21:11538. doi: 10.15766/mep_2374-8265.11538 (PMC12202713; doi:10.15766/mep_2374-8265.11538)
Supplement: Supplementary file 1 — Implementation Guide.docxPediatric Mass Casualty Incident Simulation.docxJumpSTART.docxTrauma Cognitive Aid.docxLayout for In Situ Implementation.docxDigitized Patient Templates for Distribution.docxMaterial Costs.docxPatient Presentations.docxPediatric MCI Simulation Workflow.docxSimulation Data Collection Sheet.docxPostsimulation Survey Questions.docx [file mep_2374-8265.11538-s001.zip › F. Digitized Patient Templates for Distribution.docx]

| **Appendix F: Digitized Patient Templates for Distribution** |
| --- |
| **Instructions:** Digital patients can be scaled and printed using tiled 8”x11” paper or scaled by Broselow proportions for large format print services. Each Team A will receive a unique set of 5 patients as divided below. |
| **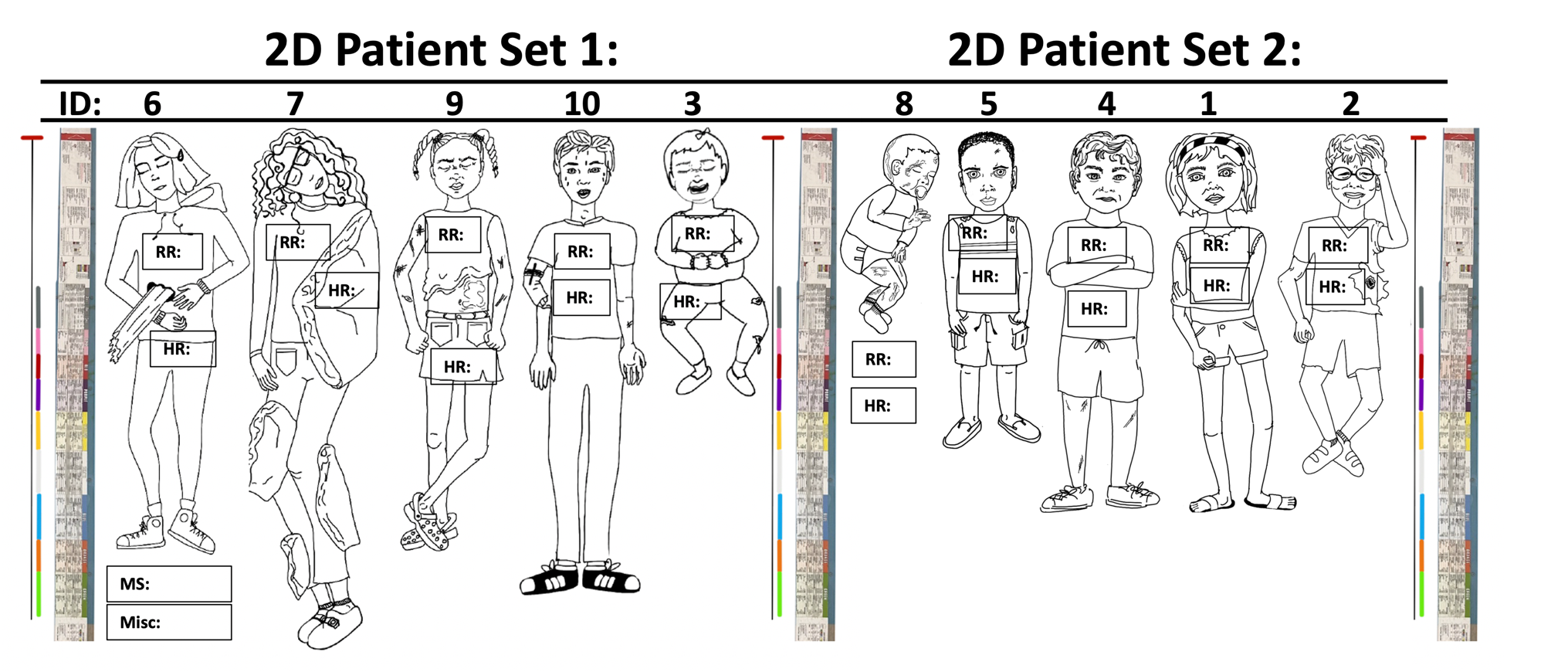**  **RR=**respiratory rate, **HR**=heart rate, **MS**=mental status |
| **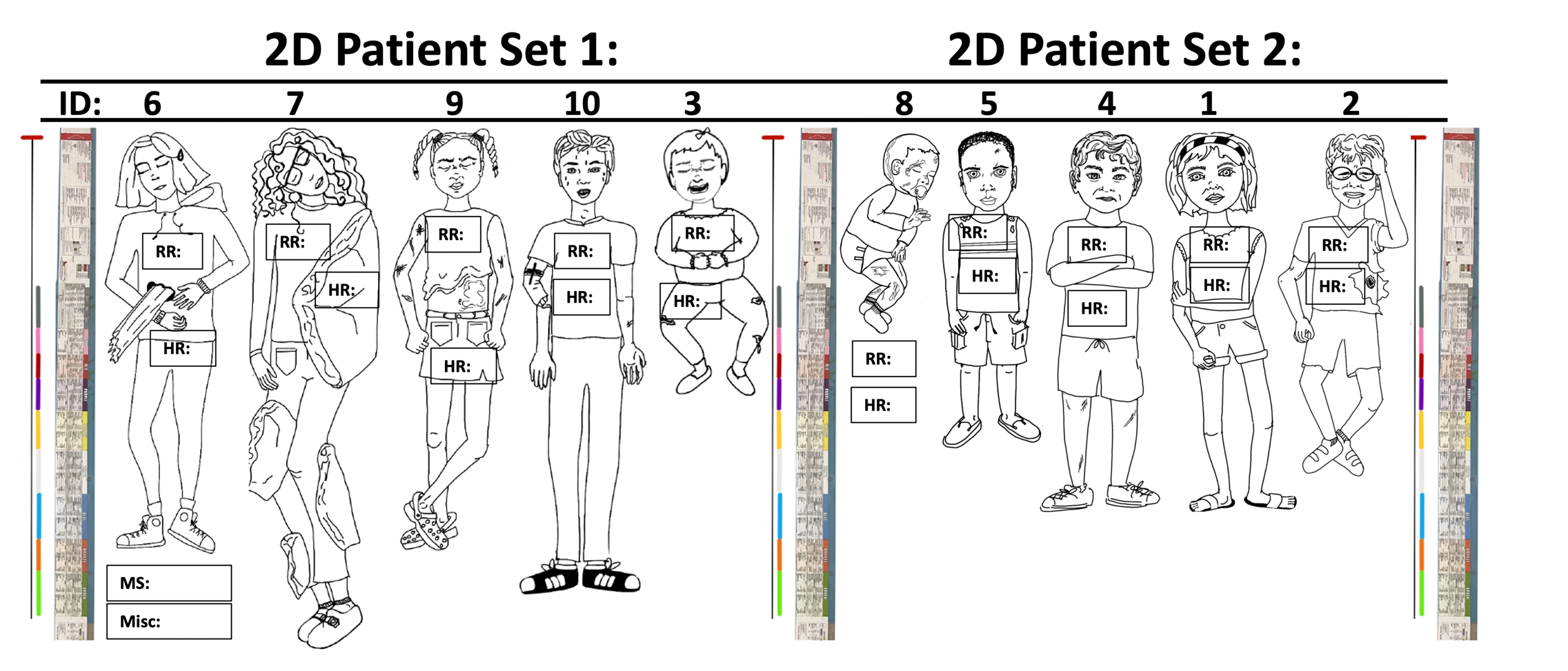**  **RR=**respiratory rate, **HR**=heart rate, **MS**=mental status  **RR, HR and other details intentionally left blank so that details of each case can be changed between simulations*  *Patient drawings are original creations of the authors and available for reproduction with citation.* |
