# Supplementary material for: Low-Fidelity, In Situ, Accessible Pediatric Mass Casualty Incident Simulation to Evaluate and Improve Pediatric Readiness
Source: MedEdPORTAL. 2025 Jun 27;21:11538. doi: 10.15766/mep_2374-8265.11538 (PMC12202713; doi:10.15766/mep_2374-8265.11538)
Supplement: Supplementary file 1 — Implementation Guide.docxPediatric Mass Casualty Incident Simulation.docxJumpSTART.docxTrauma Cognitive Aid.docxLayout for In Situ Implementation.docxDigitized Patient Templates for Distribution.docxMaterial Costs.docxPatient Presentations.docxPediatric MCI Simulation Workflow.docxSimulation Data Collection Sheet.docxPostsimulation Survey Questions.docx [file mep_2374-8265.11538-s001.zip › G. Material Costs.docx]

| Appendix G: Material Cost Breakdown | | |
| --- | --- | --- |
| **Material Item** | **Notes** | **Approximate Cost** |
| Roll of Butcher Paper | Paper utilized for 2D patient replicas | $38.00 |
| Pack of Markers | Markers utilized for drawing of 2D patient replicas | $5.00 |
| Broselow Tape | Broselow tape utilized to ensure 2D patient replicas are appropriately sized for learners to correctly assign Broselow category | $23.00 |
| JumpSTART Triage Algorithm | Cost of printing JumpSTART alorgithm poster to hang adjacent to triage area during simulation | $110.00 |
| Trauma Cognitive Aid | Cost of printing Trauma Cognitive Aid to hang adjacent to triage area during simulation | $110.00 |
| Roll of Duct Tape | Tape for hanging posters adjacent to triage area during simulation | $9.00 |
| Pack of Pens | Utilized by teams and logistic coordinators during the simulation to record data and triage decisions and next steps in management | $8.00 |
| Pack of Sticky Notes | Utilized by teams during the simulation to record triage decisions and next steps in management | $7.00 |
| **Approximate Total** **Cost** |  | **$310.00*** |

**Approximate total cost does not include cost of printing simulation orientation materials or data collection sheets*
