# Supplementary material for: Low-Fidelity, In Situ, Accessible Pediatric Mass Casualty Incident Simulation to Evaluate and Improve Pediatric Readiness
Source: MedEdPORTAL. 2025 Jun 27;21:11538. doi: 10.15766/mep_2374-8265.11538 (PMC12202713; doi:10.15766/mep_2374-8265.11538)
Supplement: Supplementary file 1 — Implementation Guide.docxPediatric Mass Casualty Incident Simulation.docxJumpSTART.docxTrauma Cognitive Aid.docxLayout for In Situ Implementation.docxDigitized Patient Templates for Distribution.docxMaterial Costs.docxPatient Presentations.docxPediatric MCI Simulation Workflow.docxSimulation Data Collection Sheet.docxPostsimulation Survey Questions.docx [file mep_2374-8265.11538-s001.zip › H. Patient Presentations.docx]

| Appendix H: Patient Presentations | | | | | | | |
| --- | --- | --- | --- | --- | --- | --- | --- |
| Instructions: Facilitators should have the patient presentations printed and available to themselves. They should be prepared to provide verbal cues to learners regarding the described status of the 2D patient if not already specified on the paper patient. | | | | | | | |
| Victim Number | Age, Sex | RR | Triage Category | Broselow Color | Perfusion | Mental status | Other |
| 1 | 7y F | 10 | Red | Blue | Distal pulse present | Groans in response to painful stimuli | Found lying down, carried in by bystander |
| 2 | 4y M | 40 | Red | White | Weak radial pulse | Withdraws from painful stimuli | Arm deformity, sucking chest wound |
| 3 | 18m F | 35 | Green | Purple | Distal pulse present | Crying | Limping, abrasions, some embedded gravel |
| 4 | 5y M | 20 | Yellow | Blue | Distal pulse present | Obeys commands | Complains they cannot move or feel their legs |
| 5 | 2y M | 28 | Red | Yellow | Distal pulse present | Not following commands | Sitting on shoulder of road, blood in ears, unwilling to walk |
| 6 | 12y F | 8 | Red | Green | Pulse absent | Unresponsive | Impaled by wooden beam |
| 7 | 17y F | 0 | Black | Brown | Weak radial pulse | Unresponsive | Trapped under rubble, apneic after five rescue breaths |
| 8 | 6m M | 0 | Black | Pink | Absent pulse | Moaning initially, now unresponsive | Found down on ground with abrasions all over his body, large occipital hematoma |
| 9 | 8y F | 40 | Yellow | Orange | Weak radial pulse | Responds to verbal stimuli | Large bruise forming on abdomen, abrasions on extremities, unable to walk |
| 10 | 13y M | 48 | Red | Brown | Rapid and weak | Blank stare | Partial amputation of R arm, diaphoretic |
