# Supplementary material for: Low-Fidelity, In Situ, Accessible Pediatric Mass Casualty Incident Simulation to Evaluate and Improve Pediatric Readiness
Source: MedEdPORTAL. 2025 Jun 27;21:11538. doi: 10.15766/mep_2374-8265.11538 (PMC12202713; doi:10.15766/mep_2374-8265.11538)
Supplement: Supplementary file 1 — Implementation Guide.docxPediatric Mass Casualty Incident Simulation.docxJumpSTART.docxTrauma Cognitive Aid.docxLayout for In Situ Implementation.docxDigitized Patient Templates for Distribution.docxMaterial Costs.docxPatient Presentations.docxPediatric MCI Simulation Workflow.docxSimulation Data Collection Sheet.docxPostsimulation Survey Questions.docx [file mep_2374-8265.11538-s001.zip › K. Postsimulation Survey Questions.docx]

| **Appendix K: Post-Simulation Survey Questions** |
| --- |
| **Instructions:** Translate the survey questions below to an anonymous digital survey or a printed handout for post-evaluation. |
| 1. Please evaluate your team's teamwork: - Communicated effectively (verbal, non-verbal, written, etc.)   **Evaluated on a 4-point Likert scale (0=Never/Hardly Ever, 1=Seldom, 2=About as often as not, 3=Often, 4=Always/Nearly Always)*   1. Please evaluate your team's teamwork: - Worked together to complete tasks in a timely manner   **Evaluated on a 4-point Likert scale (0=Never/Hardly Ever, 1=Seldom, 2=About as often as not, 3=Often, 4=Always/Nearly Always)*   1. Please evaluate your team's teamwork: - Acted with composure and control (applicable emotions? conflict management issues?)   **Evaluated on a 4-point Likert scale (0=Never/Hardly Ever, 1=Seldom, 2=About as often as not, 3=Often, 4=Always/Nearly Always)*   1. Please evaluate your team's teamwork: - Adapted to changing situations (adaptations within roles during patient deterioration or team changes)   **Evaluated on a 4-point Likert scale (0=Never/Hardly Ever, 1=Seldom, 2=About as often as not, 3=Often, 4=Always/Nearly Always)*   1. Please evaluate your team's teamwork: - Monitored and reassessed the situation   **Evaluated on a 4-point Likert scale (0=Never/Hardly Ever, 1=Seldom, 2=About as often as not, 3=Often, 4=Always/Nearly Always)*   1. Please evaluate your team's teamwork: - Anticipated potential actions (preparation of equipment, meds, etc.)   **Evaluated on a 4-point Likert scale (0=Never/Hardly Ever, 1=Seldom, 2=About as often as not, 3=Often, 4=Always/Nearly Always)*   1. Please evaluate your team's teamwork: - Prioritized tasks   **Evaluated on a 4-point Likert scale (0=Never/Hardly Ever, 1=Seldom, 2=About as often as not, 3=Often, 4=Always/Nearly Always)*   1. Please evaluate your team's teamwork: - Followed approved standards/ guidelines   **Evaluated on a 4-point Likert scale (0=Never/Hardly Ever, 1=Seldom, 2=About as often as not, 3=Often, 4=Always/Nearly Always)*   1. Give your overall rating of the team's performance - Overall Performance   *^+^Evaluated on a 10-point sliding scale (1=Poor, 10=Excellent)*   1. Have you used JumpSTART before?   *^#^Evaluated on a 10-point sliding scale (1=Not at all, 10=A great deal)*   1. Did the team use JumpSTART today?   *^#^Evaluated on a 10-point sliding scale (1=Not at all, 10=A great deal)*   1. Do you feel that JumpSTART: - Reduces variability in MCI triage assessments   *^#^Evaluated on a 10-point sliding scale (1=Not at all, 10=A great deal)*   1. Do you feel that JumpSTART: - Helps to improve efficiency during MCI events   *^#^Evaluated on a 10-point sliding scale (1=Not at all, 10=A great deal)*   1. Were there any aspects of the simulation that you found particularly educational / beneficial? 2. Do you have any additional comments, feedback, or anecdotes: |
