# Supplementary material for: Low-Fidelity, In Situ, Accessible Pediatric Mass Casualty Incident Simulation to Evaluate and Improve Pediatric Readiness
Source: MedEdPORTAL. 2025 Jun 27;21:11538. doi: 10.15766/mep_2374-8265.11538 (PMC12202713; doi:10.15766/mep_2374-8265.11538)
Supplement: Supplementary file 1 — Implementation Guide.docxPediatric Mass Casualty Incident Simulation.docxJumpSTART.docxTrauma Cognitive Aid.docxLayout for In Situ Implementation.docxDigitized Patient Templates for Distribution.docxMaterial Costs.docxPatient Presentations.docxPediatric MCI Simulation Workflow.docxSimulation Data Collection Sheet.docxPostsimulation Survey Questions.docx [file mep_2374-8265.11538-s001.zip › A. Implementation Guide.docx]

| Appendix A: Implementation Guide  **SIMULATION CASE TITLE: Pediatric Mass Casualty Incident Simulation** (*adapted from Tan et al.)*  **LEARNER AUDIENCE: Both prehospital and hospital providers including students and residents of various specialties.** | |
| --- | --- |
| This guide outlines the implementation of a pediatric mass casualty incident (MCI) simulation based on the described manuscript, designed to teach interprofessional teams the skills necessary to triage and care for pediatric patients in MCI events. | |
|  | |
| **Overview of Simulation Purpose** | Objective: To train multidisciplinary teams of healthcare providers in pediatric-specific trauma response during MCI events using the JumpSTART Pediatric Triage Algorithm and Broselow tape.  Simulation Type: Low-fidelity, multi-patient pediatric trauma simulation, replicable for frequent in-situ training. |
| **Meeting Times and Intervals** | Initial Planning Meetings:   - **Frequency**: Weekly for 1 month prior to the simulation event. - **Duration**: 60 minutes per meeting. - **Focus**: Assign responsibilities, review case scenarios, and finalize logistics.   Simulation Events:   - **Frequency**: First Wednesday of each month, during resident education time. - **Duration**: 60 minutes per session, including setup, simulation, and debriefing. |
| **Volunteers and Participants** | Volunteers: 2-4 faculty members and 2 logistics coordinators   - Roles: Facilitators, simulation setup and breakdown, data collection   Participants: Total of 16–18 per session.   - Target Audience: Surgery residents, emergency medicine residents, pediatric emergency medicine nurses, transport paramedics, emergency department technicians, respiratory therapists, students. - Participant Characteristics: Mix of novice and experienced learners in pediatric trauma and emergency care. No prerequisite knowledge required. |
| **Location** | Primary Venue: Ambulance bay of the emergency department.  Alternative Venues: Classroom, simulation lab, or any large open space that allows team movement.  Space Requirements:   - Enough space for two triage stations and 8-10 participants to move and communicate freely. - Posters of the JumpSTART Pediatric Triage Algorithm and Trauma Cognitive Aid displayed. |
| **Environmental Preparation and Event Logistics**  **Setup Time:** 15 minutes before the simulation begins. | Required Equipment:   - Two tables for triage stations. - 10 low-fidelity 2D paper models of pediatric patients (*Appendix E)*. - Broselow tapes (1 per team). - Sticky notes for recording triage categories. - Master list of patients and injury mechanisms for facilitators (*Appendix C*). - Stopwatch or phone to track time to triage. - Posters of JumpSTART Pediatric Triage Algorithm *(Appendix I*) and Trauma Cognitive Aid (*Appendix J)*. - Method to distribute post-event survey (*Appendix H)* i.e. emailed link or QR code to scan.   Triage Stations:   - Two stations, each with 5 2D patient models representing different triage categories (*Appendix D*). |
| **Volunteer Logistics** | Facilitators:   - 1 Pediatric Emergency Medicine attending and 1 Pediatric Surgeon required per session. - Responsible for participant orientation, simulation facilitation, and debriefing.   Logistics Coordinators:   - 2 coordinators to time triage, document team performance, and manage setup.   Role Preparation: Facilitators must review the JumpSTART algorithm (*Appendix I)*  and case scenarios (*Appendix C*) prior to the event. |
| **Prebriefing of Participants**  **Duration:** 10 minutes | Content:   - Briefing on the goals of the simulation and the use of the Broselow tape and JumpSTART algorithm (*Appendix I)*. - Explanation of the handoff process between the Team A (relaying team) and Team B (receiving team) (*Appendix F)* of each group respectively. - Team formation: Divide participants into groups of 8-9 within which each group will have a Team A and Team B with 4-5 participants per team (A and B). The final group structure should be as follows: **Group 1:** Team A and Team B, **Group 2:** Team A and Team B.   Instructional Tools: Display posters of the triage algorithm and trauma aid (*Appendix I, Appendix J)*. |
| **Simulation Logistics**  **Duration:** 20 minutes fort he simulation. | Simulation Workflow:   - Team A triages the patient using Broselow tape and records the findings. - Team A communicates findings to Team B, which takes over for further interventions. - Process continues for all 5 patients at each station.   Data Collection:   - Time to triage per patient. - Correct use of Broselow length and triage category. - Communication efficiency and team dynamics. |
| **Data Collection** | Metrics   - Triage category and Broselow length accuracy. - Time taken to assess and handoff each patient. - Record discrepancies in team decisions, communication, and triage outcomes.   Tools   - Stopwatch for timing. - Data sheet for recording triage results and timings (*Appendix G).*   Survey (see Debriefing): Anonymous post-event survey to gather feedback on simulation utility, team dynamics, and emotional responses. |
| **Debriefing**  **(Duration:** 15 minutes) | Key Points:   - Discuss correct and incorrect use of JumpSTART algorithm and Broselow tape. - Emotional reactions to triaging expectant (black-tag) patients. - Emphasize the need for re-triaging in dynamic MCI situations.   Facilitator-Led Discussion:   - Self-reflection on team performance and emotional impact. - Review of challenging cases and areas of team discrepancy. - Prioritization strategies for patient interventions. - Final synthesis by facilitators with practical insights and emotional preparation for MCI scenarios.   Post-Debrief Survey: Participants complete an anonymous survey for feedback (*Appendix H)*. |
| **Event Frequency and Improvement** | Intervals: Monthly simulations with varied patient scenarios to ensure repeated practice.  Continuous Improvement:   - Adjust simulation complexity based on participant feedback. - Increase simulation frequency if feasible. - Consider expanding scenarios and integrating more first responders. |
| **Total Time: 60 minutes** | Setup Time: 15 minutes before the simulation begins. Prebriefing Participants: 10 minutes Simulation Duration: 20 minutes  Debriefing Participants: 15 minutes Post-Survey: Scan QR code on exiting. |

This guide provides a comprehensive framework for the implementation of the trauma simulation, ensuring thorough training in pediatric trauma care and fostering readiness for real-world MCI events.
