# Supplementary material for: Low-Fidelity, In Situ, Accessible Pediatric Mass Casualty Incident Simulation to Evaluate and Improve Pediatric Readiness
Source: MedEdPORTAL. 2025 Jun 27;21:11538. doi: 10.15766/mep_2374-8265.11538 (PMC12202713; doi:10.15766/mep_2374-8265.11538)
Supplement: Supplementary file 1 — Implementation Guide.docxPediatric Mass Casualty Incident Simulation.docxJumpSTART.docxTrauma Cognitive Aid.docxLayout for In Situ Implementation.docxDigitized Patient Templates for Distribution.docxMaterial Costs.docxPatient Presentations.docxPediatric MCI Simulation Workflow.docxSimulation Data Collection Sheet.docxPostsimulation Survey Questions.docx [file mep_2374-8265.11538-s001.zip › B. Pediatric Mass Casualty Incident Simulation.docx]

| **Appendix B: Pediatric Mass Casualty Incident Simulation**  **SIMULATION CASE TITLE: Pediatric Mass Casualty Incident Simulation** (*adapted from Tan et al.)*  **LEARNER AUDIENCE: Both prehospital and hospital providers including students and residents of various specialties.** | |
| --- | --- |
| **PHYSICAL SETTING: Hospital ambulance bay preparing for arrival of patients** | |
| Instructions: Read this document in full prior to implementing the simulation to ensure understanding of simulation components and essential learning objectives. | |
| **Brief Narrative Description of Scenario** | You are working in the pediatric emergency department when you receive a call from EMS stating there has been an explosion at a local gathering place. You have been designated as a hospital triage officer to sort this influx of patients for priority medical treatment in the ED. Some of these patients have been triaged in the field, but it’s important to reassess the patient and perform a secondary triage designation upon their arrival in the ED. You have 10-15 minutes to triage all patients. |
| **Primary Learning Objectives** | 1. Accurately and efficiently assign triage categories to pediatric mass casualty patients using the JumpSTART triage method.  2. Effectively communicate patient information between team members to ensure consistency across the treatment team.  3. Utilize the Broselow tape to determine appropriately sized materials, medications dosages, and fluid volumes for pediatric interventions.  4. Understand the concept of triage dynamicity in a mass casualty event and accurately perform re-triage of mass casualty patients.  5. Interpret assigned triage categories to prioritize further intervention and allocate resources. |
| **Critical Actions** | 1. Utilize the JumpSTART Triage algorithm to appropriately assign a triage category to each patient.  2. Correctly utilize the Broselow tape to accurately determine appropriately sized materials, medications dosages, and fluid volumes for pediatric intervention.  3. Succinctly hand off patients to the receiving team while communicating all necessary vital information.  4. Allocate procedural and operative resources to the highest priority patients. |
| **Learner Preparation or Prework** | Learners do not need any prerequisite knowledge prior to participation in this simulation. |
