# Supplementary material for: Low-Fidelity, In Situ, Accessible Pediatric Mass Casualty Incident Simulation to Evaluate and Improve Pediatric Readiness
Source: MedEdPORTAL. 2025 Jun 27;21:11538. doi: 10.15766/mep_2374-8265.11538 (PMC12202713; doi:10.15766/mep_2374-8265.11538)
Supplement: Supplementary file 1 — Implementation Guide.docxPediatric Mass Casualty Incident Simulation.docxJumpSTART.docxTrauma Cognitive Aid.docxLayout for In Situ Implementation.docxDigitized Patient Templates for Distribution.docxMaterial Costs.docxPatient Presentations.docxPediatric MCI Simulation Workflow.docxSimulation Data Collection Sheet.docxPostsimulation Survey Questions.docx [file mep_2374-8265.11538-s001.zip › C. JumpSTART.docx]

**Appendix C: JumpSTART**

**Instructions:** The JumpSTART algorithm should be printed as a poster for display at the front of the simulation space. Alternately, the JumpSTART algorithm may be printed and distributed to Teams for reference during the triage exercise.


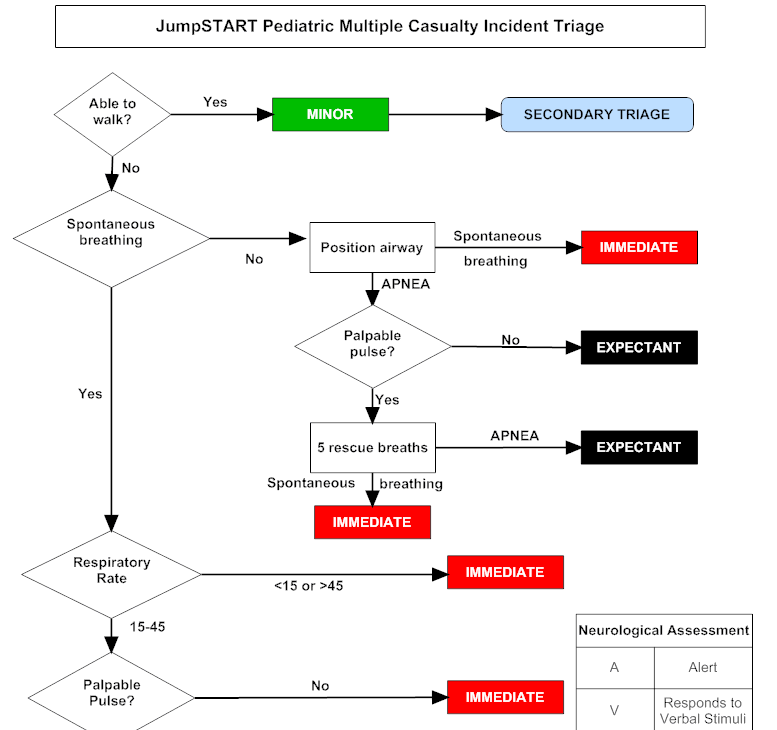

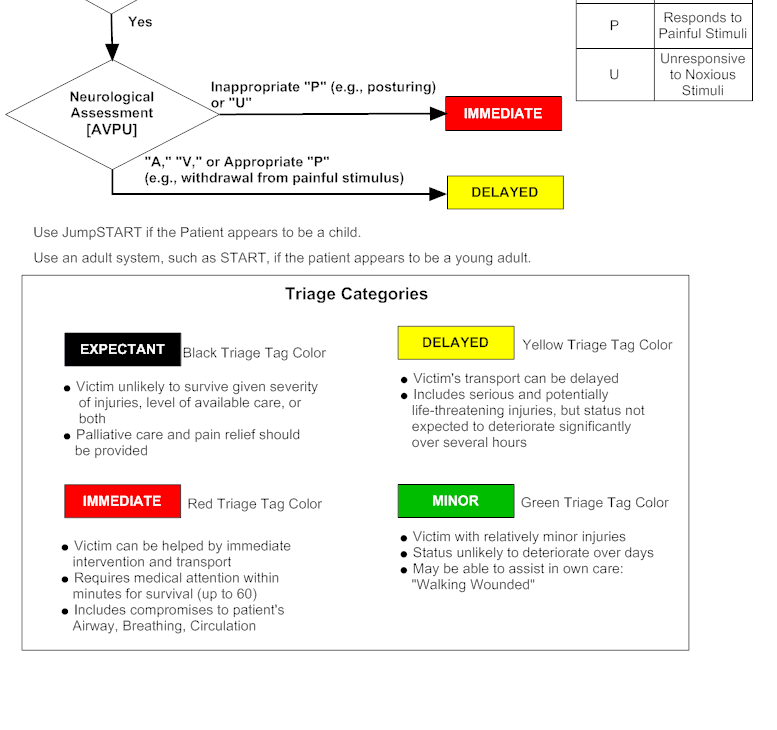

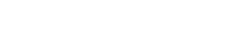


JumpSTART, a pediatric version of START, was developed at the Miami, Florida Children's Hospital in 1995 by Dr. Lou Romig.

Algorithm reproduced from the following source: “JumpSTART Pediatric Triage Algorithm - Radiation Emergency Medical Management.” *Hhs.gov*, U.S. Department of Health & Human Services, 2014, remm.hhs.gov/startpediatric.htm. Accessed 1 Sept. 2024.
