# Supplementary material for: Low-Fidelity, In Situ, Accessible Pediatric Mass Casualty Incident Simulation to Evaluate and Improve Pediatric Readiness
Source: MedEdPORTAL. 2025 Jun 27;21:11538. doi: 10.15766/mep_2374-8265.11538 (PMC12202713; doi:10.15766/mep_2374-8265.11538)
Supplement: Supplementary file 1 — Implementation Guide.docxPediatric Mass Casualty Incident Simulation.docxJumpSTART.docxTrauma Cognitive Aid.docxLayout for In Situ Implementation.docxDigitized Patient Templates for Distribution.docxMaterial Costs.docxPatient Presentations.docxPediatric MCI Simulation Workflow.docxSimulation Data Collection Sheet.docxPostsimulation Survey Questions.docx [file mep_2374-8265.11538-s001.zip › D. Trauma Cognitive Aid.docx]

***Instruction:*** *This aid should be printed or displayed for participants to reference during the simulation exercise.*

**Appendix D: Trauma Cognitive Aid**

**Trauma Resuscitation Aids**

Structured Observation Tool for Trauma Resuscitation

| **Pre-Arrival** |
| --- |
| ⬜ Activate trauma  ⬜ Confirm team roles and lead  ⬜ Team role labels and introduction  ⬜ PPE  ⬜ Crown control |
| ⬜ **Brief team: age/mechanism/injuries** |
| ⬜ Estimated weight: kg or Broselow color  ⬜ Broselow on bed  ⬜ Zero bed  ⬜ Warm room |
| ⬜ **Notify specialty service(s)** |
| **Equipment**  ⬜ Lab draw supplies  ⬜ Manual BP cuff and set monitor BP cycle  ⬜ Stethoscope  ⬜ Ultrasound machine  ⬜ Intubation equipment and med prep  ⬜ Mechanism/injury/size specific equipment  ⬜ Blood product prep and attending signs form  ⬜ Warming device if appropriate |
| ⬜ **START TIMER**  ⬜ **EMS MISTS (30 sec.)** |

| Primary Survey Verbalize | | |
| --- | --- | --- |
| A | Airway protected and patent? | ⬜ C-spine immobilized |
|  | If airway not secure:  ⬜ Verbalize airway backup plan  ⬜ GCS assessed before giving RSI medications  ⬜ Secure airway, place OG  ⬜ Report Et-tube size and depth  ⬜ ET-CO2 reading  ⬜ CXR to confirm placement | |
| B | ⬜ O2 if sat<90%  ⬜ Bilateral breath sounds | ⬜ Absent breath sounds and unstable? Place chest tube |
| C | ⬜ Central/distal pulses and BP  ⬜ 2 Large bore IV/IO  ⬜ Labs drawn and ordered | Pediatric:  PRBC: 20 cc/kg  FFP: 10 cc/kg  Platelets: 5 cc/kg  If > 40 cc/kg consider  MTP 1:1:1 |
|  | **Consider:**  ⬜ Chest-XR, Pelvis-XR, e-FAST  ⬜ CVC  ⬜ Blood products  ⬜ Pelvic binder and hemorrhage control |  |
| D | ⬜ GCS (eyes 1-4, verbal 1-5, motor 1-6)  ⬜ Pupil size/response | |
| E | ⬜ Remove clothing  ⬜ Check temperature and warm patient | |
| v/s | ⬜ Obtain repeat BP  ⬜ Verbalize any abnormal vital signs | |
| FIRST PAUSE  ⬜ Ensure quiet and crowd control  ⬜ Summarize primary survey findings  ⬜ Ensure emergent procedures complete  ⬜ Blood transfusion status update  ⬜ Trauma order-set signed | | |
| ***“Any objections to moving on to the secondary survey?”*** | | |

| **Secondary Survey** |
| --- |
| **Verbalize findings for:**  ⬜ Head  ⬜ Face  ⬜ Ears  ⬜ C-spine/Neck  ⬜ Chest  ⬜ Abdomen  ⬜ Pelvis  ⬜ Lower extremities  ⬜ Upper extremities  ⬜ Neuro exam  ⬜ Log roll: back/rectum |
| ⬜ Obtain height and weight |
| **SECOND PAUSE**  ⬜ Summarize primary and secondary findings  ⬜ Ensure emergent procedures complete  ⬜ Chest/Pelvic x-ray  ⬜ Clarify: analgesia, antibiotics, tetanus  ⬜ Ensure labs sent  ⬜ Read back VBG/Shock if available  ⬜ Page required for consults |
| ⬜ **Communicate care plan to team (CT/IR/OR/ICU)** |

| **Prepare for Travel** |
| --- |
| ⬜ ID band on patient  ⬜ Equipment/medications/blood  ⬜ Secure lines and tubes  ⬜ Apply warm blanket  ⬜ Identify who will travel with patient  ⬜ Discuss disposition  ⬜ Prepare post-imaging supplies: foley, C-collar, tetanus, meds etc. |

Trauma Aid adapted by the author group from: Thornton, S. W., et al. (2022). "Use of a Structured Observation Tool to Promote Medical Student Engagement in Trauma Resuscitations." J Surg Educ 79(6): 1422-1425.
