# Supplementary material for: Low-Fidelity, In Situ, Accessible Pediatric Mass Casualty Incident Simulation to Evaluate and Improve Pediatric Readiness
Source: MedEdPORTAL. 2025 Jun 27;21:11538. doi: 10.15766/mep_2374-8265.11538 (PMC12202713; doi:10.15766/mep_2374-8265.11538)
Supplement: Supplementary file 1 — Implementation Guide.docxPediatric Mass Casualty Incident Simulation.docxJumpSTART.docxTrauma Cognitive Aid.docxLayout for In Situ Implementation.docxDigitized Patient Templates for Distribution.docxMaterial Costs.docxPatient Presentations.docxPediatric MCI Simulation Workflow.docxSimulation Data Collection Sheet.docxPostsimulation Survey Questions.docx [file mep_2374-8265.11538-s001.zip › E. Layout for In Situ Implementation.docx]

| **Appendix E: Layout for *in situ* Implementation** |
| --- |
| **Instructions:** Utilize the suggested layout to organize teams, instructional diagrams, and distribute facilitators across the room. |
| **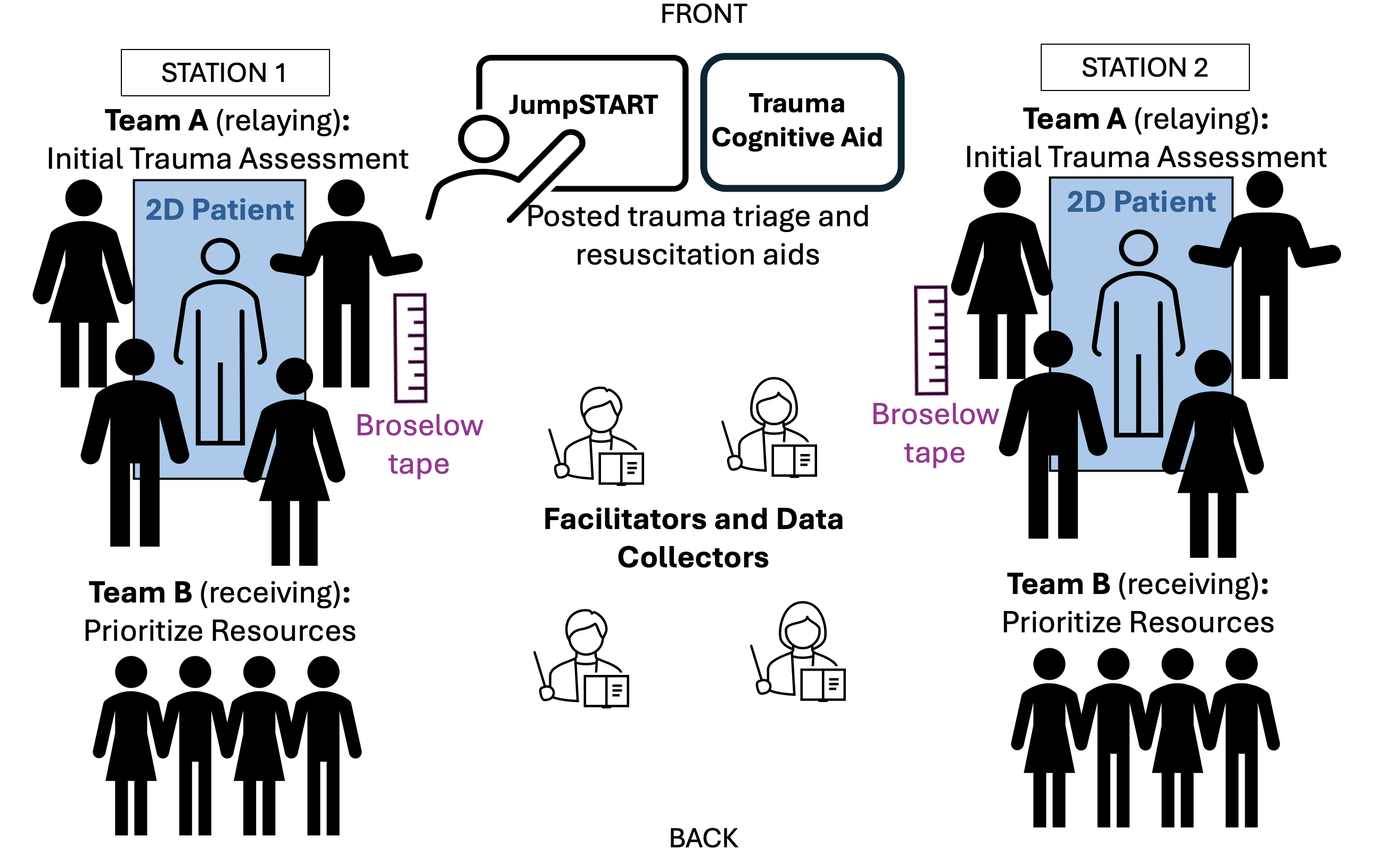** |
| **This is an author created image.* |
